# Supplementary material for: Established and Novel Risk Factors for 30-Day Readmission Following Total Knee Arthroplasty: A Modified Delphi and Focus Group Study to Identify Clinically Important Predictors
Source: J Clin Med. 2023 Jan 17;12(3):747. doi: 10.3390/jcm12030747 (PMC9917714; doi:10.3390/jcm12030747)
Supplement: Supplementary file 1 [file jcm-12-00747-s001.zip › jcm-2128330-supplementary.pdf]

Table of Contents

Qualtrics survey 2
Focus group worksheet 18
Table S1: Vote counts for each Delphi risk factor 21
Table S2: Consensus calculations for Delphi survey 22
Table S3: Risk factors suggested by Delphi survey participants 24
Table S4: Vote counts for each focus group risk factor 25
Table S5: Focus group (initial vote) risk factors with a majority vote (0.5 threshold) of high importance 27
Table S6: Reasons given for changes in risk factor votes 30
Table S7: Consensus calculations for focus group 34
Figure S1: Counts of votes - Focus group initial votes 35
Figure S2: Focus group thematic analysis coding tree 36

Qualtrics survey

Risk factors for 30-day readmission after primary total knee replacement

Survey Flow

Block: Risk factors for 30-day readmission after primary total knee replacement (2 Questions)
Standard: Participant information (3 Questions)
Standard: Block 6 (1 Question)
Standard: Factors from systematic review and meta-analysis (1 Question)
Standard: Factors in the St Vincent's hospital administrative system (2 Questions)
Standard: Other comments (1 Question)

Page Break

Start of Block: Risk factors for 30-day readmission after primary total knee replacement

Q1 SUMMARY EXPLANATION OF RESEARCH

Title: Predictive Factors for 30-Day Readmission Following Total Knee Arthroplasty – A Modified Delphi Survey Study

Principal investigator: Professor Peter Choong
Study Co-ordinator: Daniel Gould

The objectives of this study are to (1) present to clinicians of varying levels of expertise the best available evidence for risk factors for 30-day readmission following TKA such that they can appraise each individual risk factor and utilise their clinical training and experience to judge the impact of each risk factor on readmission risk, and (2) provide these clinicians with the opportunity to suggest different risk factors which were not previously identified.

We are inviting clinicians involved in the care of total knee arthroplasty (TKA) patients to complete a one-off anonymous survey to draw on clinical knowledge and experience in order to identify risk factors for readmission after primary TKA. The time required to complete this survey is less than 10 minutes. There are no costs associated with participating in this research project, nor will you be paid. Identifying details are not collected, so the survey is anonymous.

Your clinical insight is important as it will help us determine which risk factors are most important for predicting readmission in primary TKA patients, including suggestions from participating clinicians regarding risk factors we had not previously encountered in the systematic review and meta-analysis on this topic. This will inform the development of a clinical machine learning risk prediction model for 30-day readmission in primary TKA patients. The goal is to achieve consensus on the relevance and strength risk factors included in this survey. Any risk factors for which consensus is not reached after the two-week period for which the survey is open will be discussed in person between two representatives at St Vincent's Hospital Melbourne from each group we are recruiting into this study, i.e. two consultant orthopaedic surgeons, two consultant anaesthetists, two orthopaedic registrars, and two orthopaedic residents. The invitation to participate in this discussion will be sent to the same St Vincent's Hospital Melbourne clinicians invited to participate in this survey. This session will be coordinated by the study co-ordinator, Daniel Gould.

Commencing this questionnaire implies your voluntary consent to participate in the research. Participation is completely voluntary and you may end your participation at any time. All responses will be kept anonymous. All information will be held in the strictest of confidence. Results from the research will be reported as aggregate data.

Participating sites: St Vincent's Hospital Melbourne; Western Health; Northern Health; Austin Health; Box Hill Hospital; Bendigo Health; Ballarat Health Service; Barwon Health; Eastern Health (Box Hill Hospital); Goulburn Valley Health; Wangaratta Hospital

Further information and who to contact regarding further information and complaints, should any arise:

Contact person Name: Daniel Gould
Position: Study Coordinator
Telephone:
Email:

Thank you in advance for your time.

Q2 Do you consent to participate? (answering 'No' will submit this form and prevent you from progressing to view the clinical cases)

- ○ Yes (1)
- ○ No (2)

End of Block: Risk factors for 30-day readmission after primary total knee replacement

---

Start of Block: Participant information

Display This Question:

If Q2 = 1

Q3 What is your designation?

○ Consultant orthopaedic surgeon (1)

○ Consultant anaesthetist (2)

○ Orthopaedic registrar (3)

○ Orthopaedic resident (4)

Q4 How long have you been at this level? (answer in number of years)

____

Q5 What is your case load (approximate number of TKA patients you see per year. There is no minimum case load - it can be zero)?

____

End of Block: Participant information

Start of Block: Block 6

Q6 Based on your clinical knowledge and experience, please list as many risk factors for 30-day readmission following primary TKA as you can think of. These can be patient-related or non-patient-related, and you can assume the information would be readily available (i.e. you do not need to consider the logistical or other challenges involved in obtaining the information). Please refrain from altering this list after you move on to the next page. You will be given more opportunities to suggest risk factors throughout the survey, but the purpose of this section is to ascertain your thoughts prior to seeing the rest of the survey.

____

End of Block: Block 6

Start of Block: Factors from systematic review and meta-analysis

Q7 Based on your clinical knowledge and experience, what is the importance of each of the following risk factors when considering a patient's overall risk of readmission within 30 days following primary TKA? There was moderate or high quality evidence for the effect of these risk factors in systematic review and meta-analysis. Take this information into account, but please consider your own clinical impression and experience.<br>Each risk factor is presented as odds ratio (95% confidence interval) - or narrative synthesis findings - and quality of evidence as per GRADE criteria:<br>- GRADE +++ moderate quality = moderately confident in the effect estimate: true effect is likely to be close to the estimate of the effect, but there is a possibility that it is substantially different;<br>- GRADE ++++ high quality = very confident that the true effect lies close to that of the estimate of the effect.<br>If odds ratio and 95% confidence interval are greater than 1, this indicates	Low importance (1)	Moderate importance (2)	High importance (3)

increased readmission risk. If you would like more detail on any particular risk factor, here is a link to open-access manuscript: https://www.mdpi.com/2077-0383/10/1/134.			
Alcohol abuse (meta-analysis: 1.08 (0.96–1.20), ++++) (1)	○	○	○
BMI (mixed findings. Best evidence = narrative synthesis: 1/2 univariate analyses found increased risk; 0/1 multivariate analyses found increased risk, +++) (6)	○	○	○
Arrhythmia (meta-analysis: 1.14 (1.09–1.19), +++) (7)	○	○	○
Peripheral vascular disease (meta-analysis: 1.17 (1.10–1.24), +++) (8)	○	○	○
Diabetes - NIDDM (meta-analysis: 1.08 (0.80–1.45), +++) (9)	○	○	○
Liver disease (meta-analysis: 1.29 (1.20–1.39), +++) (10)	○	○	○
Peptic ulcer disease (meta-analysis: 0.94 (0.84–1.07), +++) (11)	○	○	○

Anaemia (meta-analysis: 1.19 (1.15–1.24), ++++) (12)			
Coagulopathy (meta-analysis: 1.25 (1.15–1.36), +++) (13)			
Fluid and electrolyte disorder (meta-analysis: 1.05 (1.00–1.12), +++) (14)			
Paralysis (meta-analysis: 1.13 (0.97–1.31), +++) (15)			
Smoking (meta-analysis: 1.25 (0.82–1.91), +++) (16)			
Charlson Comorbidity Index ≥1 (narrative synthesis: majority of multivariate analyses found increased risk, studies were +++ or ++++) (17)			
Hypertension (narrative synthesis: 2/4 univariate analyses and 6/7 multivariate analyses found increased risk, +++) (18)			
Hyperlipidaemia (narrative synthesis: 1/2 univariate analyses found increased risk, +++) (19)			

Congestive heart failure (CHF) (narrative synthesis: 3/3 univariate analyses and 5/7 multivariate analyses found increased risk) (20)	○	○	○
Recent weight loss (narrative synthesis: 0/1 univariate analysis and 0/1 multivariate analysis found increased risk, +++) (21)	○	○	○
Hypothyroidism (narrative synthesis: 0/1 univariate analysis and 0/1 multivariate analysis found increased risk, +++) (22)	○	○	○
Elevated serum BUN (narrative synthesis: 1/1 univariate analysis and 1/1 multivariate analysis found increased risk, +++) (23)	○	○	○
Low serum albumin (narrative synthesis: 1/1 multivariate analysis found increased risk, +++) (24)	○	○	○
Pulmonary disease (narrative synthesis: 1/1 univariate analysis and 1/2 multivariate analyses found increased risk, +++) (25)	○	○	○
Depression (narrative synthesis: 1/3 univariate analyses and 3/4 multivariate analyses found increased risk, +++) (26)	○	○	○

Substance abuse (narrative synthesis: 1/2 univariate analyses and 2/2 multivariate analyses found increased risk, +++) (27)	○	○	○
History of cancer (narrative synthesis: 1/1 univariate analysis and 2/3 multivariate analyses found increased risk (28)	○	○	○
Previous stroke (narrative synthesis: 0/1 univariate analysis, 1/2 multivariate analyses found increased risk, 1/2 found reduced risk, +++) (29)	○	○	○
Dementia (narrative synthesis: 1/1 multivariate analysis found increased risk, +++) (30)	○	○	○
In-hospital complication (any) (narrative synthesis: 1/1 univariate analysis and 1/1 multivariate analysis found increased risk, ++++) (31)	○	○	○
Chronic kidney disease (CKD) (narrative synthesis: 1/1 univariate analysis and 2/3 multivariate analyses found increased risk, +++) (32)	○	○	○

Wound class (narrative synthesis: 1/1 univariate analysis and 0/2 multivariate analyses found increased risk, +++) (33)	○	○	○
Race (narrative synthesis: 2/2 univariate analyses and 1/1 multivariate analysis found increased risk based on racial differences, +++) (34)	○	○	○
Age (narrative synthesis: 3/4 univariate analyses and 4/7 multivariate analyses found increased risk with differences in age, +++) (35)	○	○	○
Male sex (narrative synthesis: 2/4 univariate analyses and 8/8 multivariate analyses found increased risk, ++++) (36)	○	○	○
Low socioeconomic status (narrative synthesis: 1/1 univariate analysis and 1/1 multivariate analysis found increased risk, +++) (37)	○	○	○
Dependent functional status (narrative synthesis: 1/1 multivariate analysis found increased risk, +++) (38)	○	○	○
Elective surgery (vs urgent) (meta-analysis: 0.82 (0.71–0.95), +++) (39)	○	○	○

Increasing number of previous admissions (narrative synthesis: 2/2 multivariate analyses found increased risk, +++) (40)	○	○	○

End of Block: Factors from systematic review and meta-analysis

Start of Block: Factors in the St Vincent's hospital administrative system

Q8 Based on your clinical knowledge and experience, what is the importance of each of the following risk factors when considering a patient's overall risk of readmission within 30 days following primary TKA?
These factors are available from hospital administrative database or are collected directly from patients undergoing primary TKA at St Vincent's.

	Low importance (1)	Moderate importance (2)	High importance (3)
Preoperative patient-reported level of function (1)	○	○	○
Preoperative patient-reported pain level (2)	○	○	○
Preoperative patient-reported quality of life (3)	○	○	○
Seniority of the operating surgeon (4)	○	○	○
Type of anaesthetic (5)	○	○	○
Transfusion during surgery (6)	○	○	○
Details on implants used (7)	○	○	○
Duration of operation (8)	○	○	○
Time of operation (i.e. time in the day) (9)	○	○	○
ICU/HDU admission (10)	○	○	○

Length of stay (11)	○	○	○
Return to theatre (12)	○	○	○
Rehab stay (13)	○	○	○
Aboriginality (14)	○	○	○

Q9 Are there any other factors (other than those listed above) that you think predict readmission within 30-days of primary TKA? These can be patient-related or non-patient-related, and you can assume the information would be readily available (i.e. you do not need to consider the logistical or other challenges involved in obtaining the information).
If yes, please list ALL other factors and indicate whether they are strong, moderate, or weak. If no, please state "no".
Examples include, but are not limited to (Miller 2018 - DOI 10.1186/s12913-018-2833-3):
- patients identified as having poor understanding of their disease,
- having poor adherence to therapy, having poor social support or access to care,
- having a condition that is likely to relapse or worsen,
- requiring therapy that is likely to result in a complication,
- having a high likelihood of developing a new medical condition that would require readmission,
- having an organ transplant,
- having a previous readmission.

____

End of Block: Factors in the St Vincent's hospital administrative system

---

Start of Block: Other comments

Q10 Please provide any other comments on survey design, risk prediction, comments about specific risk factors, or any other relevant topic.

____

End of Block: Other comments

Focus group worksheet

Predictive Factors for 30-Day Readmission Following Total Knee Arthroplasty – A Modified Delphi Survey and Focus Group Study

About you:

Study ID:

How long have you been at your current designation (consultant orthopaedic surgeon, consultant anaesthetist, orthopaedic registrar)?

What is your case load (approximate number of TKA patients you see per year)? (This can be zero)

Objective of this study:

The risk factors to be discussed in this study were suggested in a previous Delphi study completed by more than 30 of your colleagues. These particular risk factors were selected for discussion because there is little to no evidence in the literature regarding their impact on risk of 30-day readmission for TKA patients.

The purpose of this study is to utilise your expertise and experience in a systematic way to gain some level of clinical judgement on the importance of each risk factor for inclusion in a multivariable risk prediction model for 30-day readmission in TKA patients. Assume the information is readily available, so you can focus on the predictive importance of each risk factor rather than technical or logistical concerns.

How to complete the table:

Simply mark with 'X' your initial vote, and then mark your vote after discussion of the risk factor if you changed your vote.

	**Initial vote**	**Vote after discussion (if changed)**

**Risk factors**	Low importance	Moderate importance	High importance	Low importance	Moderate importance	High importance
High risk of infection: immunocompromised state, active IVDU, infection in other primary joint replacement						
Poor access to post-op care: lives far from hospital, lack of access to allied health support, lack of access to telehealth support						
Inadequate pain management at discharge						
Surgical factors: prolonged/complex/difficult surgery, surgical misadventure						
Poor understanding of disease and post-op course						
Inexperienced surgeon/specific surgeon						
Patient-related biopsychosocial: lower education level, poor health literacy, non-English speaking						
Poor compliance with rehab						
Pain catastrophizing; analgesia intolerance/catastrophic pain						
Resilience						

Previous readmission (e.g. with prior contralateral TKA)						
Haemoglobin - pre-op and on discharge						
Delayed surgery						
Inadequate nursing ratios						
Threshold for readmission - e.g. the specific ED, or whether there is a junior registrar reviewing the patient						
Multi-joint disease						
Personality disorders						
Poor skin condition						
Osteoporosis						
Transplant recipient (better at self-managing medications, better screened for other comorbidities, etc.)						
Patients who do not actually want the surgery but have been pressured by family, doctors etc to undergo the procedure.						
Ethnic minorities. In the U.S. - Black population. In Australia- Aboriginal and Torres Strait Islander Peoples, Refugees, Homelessness						
IVDU = intravenous drug use; TKA = total knee arthroplasty; ED = emergency department; U.S. = United States						

Table S1: Vote counts for each Delphi risk factor

**Risk factor**	**Number of 'low importance' votes**	**Number of 'moderate importance' votes**	**Number of 'high importance' votes**
*Return to theatre	0	9	27
Any in hospital complication	0	12	24
*ICU or HDU admission	1	11	24
Dependent functional status	4	11	20
Dementia	4	13	19
*Preoperative patient reported pain level	2	15	19
Liver disease	4	13	18
Charlson Comorbidity Index	3	13	18
Substance abuse	3	14	18
Increasing number of previous admissions	1	16	18
CHF	4	16	16
Paralysis	9	13	13
**Anaemia	3	27	6
Wound class	5	22	8
Elevated serum BUN	9	22	3
**Low socioeconomic status	6	21	8
Rehab stay	8	21	7
Aboriginality	11	21	3
Length of stay	3	20	13
**CKD	4	20	11
Duration of operation	8	20	8
**Coagulopathy	3	19	14
Low serum albumin	4	19	13
Transfusion during surgery	7	19	10
Pulmonary disease	7	19	9
Alcohol abuse	5	18	13
**Depression	11	18	7
Previous stroke	10	18	7

Preoperative patient reported level of function	3	17	16
**Age	6	17	13
**Arrhythmia	8	17	10
Smoking	10	17	9
**History of cancer	16	17	2
**Peripheral vascular disease	5	16	15
NIDDM	5	16	14
BMI	6	16	14
Preoperative patient reported quality of life	8	14	13
Hyperlipidaemia	30	6	0
Details on implants used	27	7	1
Peptic ulcer disease	26	8	2
**Hypertension	26	9	1
Hypothyroidism	26	9	0
Type of anaesthetic	24	12	0
**Race	22	13	1
Time of operation	21	13	2
Recent weight loss	19	15	1
Fluid and electrolyte disorder	18	12	6
**Male sex	18	14	3
Elective surgery vs urgent	17	13	5
Seniority of the operating surgeon	16	15	5

ICU = intensive care unit; HDU = high dependency unit; CHF = congestive heart failure; BUN = blood urea nitrogen; CKD = chronic kidney disease; NIDDM = non-insulin-dependent diabetes mellitus; BMI = body mass index; * = majority (≥50%) voted as high-importance despite lack of systematic review evidence for the risk factor correlating with readmission; ** = majority did not vote as high-importance despite systematic review evidence available indicating this is a risk factor which correlates with readmission

Table S2: Consensus calculations for Delphi survey

**Study sample**	**Krippendorff's alpha (95% confidence interval)**
All participants	0.0435 (0.0151 to 0.0705)
0.5 threshold*, high importance	-0.0042 (-0.0210 to 0.0104)
0.5 threshold, moderate importance	-0.0051 (-0.0192 to 0.0074)

0.5 threshold, low importance	-0.0015 (-0.0159 to 0.0104)
2/3 threshold, high importance	-0.0122 (-0.019 to -0.0112)
2/3 threshold, moderate importance	Unable to calculate (only one observation)
2/3 threshold, low importance	-0.0064 (-0.0219 to 0.0054)
Consultant anaesthetists	0.0251 (0.0022 to 0.0465)
Consultant orthopaedic surgeons	0.0226 (-0.0168 to 0.0623)
Orthopaedic registrars	0.2711 (0.0540 to 0.4681)
All orthopaedic participants (combined category: consultants and registrars)	0.0425 (-0.0039 to 0.0921)
All participants – only risk factors with high-quality evidence according to GRADE approach systematic review and meta-analysis	0.0817 (-0.0082 to 0.1468)
All participants – only risk factors with moderate-quality evidence according to GRADE approach systematic review and meta-analysis	0.0204 (-0.0011 to 0.0402)
*threshold refers to the proportion of votes in that category, e.g. '0.5 threshold, high importance' means the calculation for Krippendorff's alpha was restricted to risk factors for which at least half the votes were in the 'high importance' category; GRADE = Grading of Recommendations Assessment, Development and Evaluation	

Table S3: Risk factors suggested by Delphi survey participants

Risk factor	Number of participants who independently suggested the risk factor
High risk of infection (immunocompromised state, active IVDU, infection in other primary joint replacement)	12
Poor access to post-op care (lives far from hospital, lack of access to allied health support, lack of access to telehealth support)	10
Inadequate pain management at discharge	6
Surgical factors (prolonged/complex/difficult surgery, surgical misadventure)	6
Poor understanding of disease and post-op course	7
Inexperienced surgeon/specific surgeon	4
Patient-related biopsychosocial (lower education level, poor health literacy, non-English speaking)	4
Poor compliance with rehabilitation	3
Pain catastrophizing or analgesia intolerance/catastrophic pain	2
Resilience	2
Previous readmission (e.g. with prior contralateral TKA)	2
Haemoglobin (pre-op and on discharge)	2
Delayed surgery	1
Inadequate nursing ratios	1
Threshold for readmission (e.g. the specific Emergency Department, or whether there is a junior registrar reviewing the patient)	1
Multi-joint disease	1
Personality disorders	1
Poor skin condition	1
Osteoporosis	1
Transplant recipient (better at self-managing medications, better screened for other comorbidities, etc.)	1
Pressured into surgery (patients who do not actually want the surgery but have been pressured by family, doctors etc to undergo the procedure)	1
Ethnic minorities (In the US: black population. In Australia: Aboriginal and Torres Strait Islander Peoples, Refugees, Homelessness)	1
IVDU = intravenous drug use; TKA = total knee arthroplasty	

Table S4: Vote counts for each focus group risk factor

Risk factor	Number of 'low importance' votes	Number of 'moderate importance' votes	Number of 'high importance' votes
High risk of infection: immunocompromised state, active IVDU, infection in other primary joint replacement		1 → 2	5 → 4
Poor access to post-op care: lives far from hospital, lack of access to allied health support, lack of access to telehealth support	1	2 → 4	3 → 1
Inadequate pain management at discharge	1 → 0	1 → 3	4 → 3
Surgical factors: prolonged/complex/difficult surgery, surgical misadventure		2 → 3	4 → 3
Poor understanding of disease and post-op course	4 → 5	1	1 → 0
Inexperienced surgeon/specific surgeon	4	2	
Patient-related biopsychosocial: lower education level, poor health literacy, non-English speaking	1	4	1
Poor compliance with rehab	2 → 1	3 → 5	1 → 0
Pain catastrophizing; analgesia intolerance/catastrophic pain			6
Resilience		4 → 5	2 → 1
Previous readmission (e.g. with prior contralateral TKA)	3 → 4	3 → 2	
Haemoglobin - pre-op and on discharge	3 → 4	3 → 2	
Delayed surgery	6 → 5	0 → 1	
Inadequate nursing ratios	5 → 6	1 → 0	
Threshold for readmission - e.g. the specific ED, or whether there is a junior registrar reviewing the patient	3 → 0	2 → 3	1 → 3
Multi-joint disease	4 → 5	1	1 → 0
Personality disorders		5	1
Poor skin condition		6	
Osteoporosis	4 → 5	2 → 1	

Transplant recipient (better at self-managing medications, better screened for other comorbidities, etc.)		2	4
Patients who do not actually want the surgery but have been pressured by family, doctors etc to undergo the procedure	6		
Ethnic minorities. In the U.S. - black population. In Australia, Aboriginal and Torres Strait Islander Peoples. Refugees. Homelessness	1 → 2	3	2 → 1
Arrow (→) depicts change from initial vote to final vote following discussion; IVDU = intravenous drug use; TKA = total knee arthroplasty; ED = emergency department; U.S. = United States			

Table S5: Focus group (initial vote) risk factors with a majority vote (0.5 threshold) of high importance

**Groups:**	**Everyone**	**Consultant anaesthetists**	**Consultant orthopaedic surgeons**	**Orthopaedic registrars**	**All orthopaedic participants**
**Risk factors (proportion of votes in high importance category):**	Pain catastrophizing analgesia intolerance catastrophic pain (1.00)	High risk of infection immunocompromised state active IVDU infection in other primary joint replacement (1.00)	Inadequate pain management at discharge (1.00)	High risk of infection immunocompromised state active IVDU infection in other primary joint replacement (1.00)	Pain catastrophizing analgesia intolerance catastrophic pain (1.00)
	High risk of infection immunocompromised state active IVDU infection in other primary joint replacement (0.83)	Poor access to post op care lives far from hospital lack of access to allied health support lack of access to telehealth support (1.00)	Surgical factors prolonged complex difficult surgery surgical misadventure (1.00)	Pain catastrophizing analgesia intolerance catastrophic pain (1.00)	High risk of infection immunocompromised state active IVDU infection in other primary joint replacement (0.75)
	Inadequate pain management at discharge (0.67)	Surgical factors prolonged complex difficult surgery surgical misadventure (1.00)	Pain catastrophizing analgesia intolerance catastrophic pain (1.00)	Inadequate pain management at discharge (0.50)	Inadequate pain management at discharge (0.75)
	Surgical factors prolonged complex difficult surgery surgical misadventure (0.67)	Pain catastrophizing analgesia intolerance catastrophic pain (1.00)	High risk of infection immunocompromised state active IVDU infection in other primary joint replacement (0.50)	Transplant recipient better at self-managing medications better screened for other comorbidities etc (0.50)	Surgical factors prolonged complex difficult surgery surgical misadventure (0.50)
	Transplant recipient better at self-managing medications better screened for other comorbidities etc (0.67)	Resilience (1.00)	*Poor access to post op care lives far from hospital lack of access to allied health support lack of access to telehealth support (0.50)		Transplant recipient better at self-managing medications better screened for other comorbidities etc (0.50)

	*Poor access to post op care lives far from hospital lack of access to allied health support lack of access to telehealth support (0.50)	Transplant recipient better at self-managing medications better screened for other comorbidities etc (1.00)	Threshold for readmission e.g. the specific ED or whether there is a junior registrar reviewing the patient (0.50)		
		*Inadequate pain management at discharge (0.50)	Personality disorders (0.50)		
		*Poor understanding of disease and post op course (0.50)	Transplant recipient better at self-managing medications better screened for other comorbidities etc (0.50)		
		Patient related biopsychosocial lower education level poor health literacy non-English speaking (0.50)	Ethnic minorities - In the US: Black population; In Australia: Aboriginal and Torres Strait Islander Peoples, Refugees, Homelessness (0.50)		
		*Poor compliance with rehab (0.50)			
		*Multi joint disease (0.50)			
		*Ethnic minorities In the US Black population In Australia: Aboriginal and Torres Strait Islander Peoples; Refugees Homelessness (0.50)			

*Moved out of high importance category through subsequent discussion (therefore present in this table but absent from Table 3 (main text)); IVDU = intravenous drug use; ED = emergency department;

Table S6: Reasons given for changes in risk factor votes

Risk factor	Discussion of risk factor
High risk of infection: immunocompromised state, active IVDU, infection in other primary joint replacement	Not captured (recording failure)
Poor access to post-op care: lives far from hospital, lack of access to allied health support, lack of access to telehealth support	**Study ID 4 (consultant orthopaedic surgeon): "**Yeah I guess I thought that it wasn't of no importance, but there were probably other things that were of higher importance. So, it may impede patient recovery, but it's unlikely to put them back in hospital."
Inadequate pain management at discharge	**Study ID 9 (consultant anaesthetist): "**I had low importance because they've been discharged, so to be able to be discharged, they would have had an analgesic plan which if, even if it wasn't 100 per cent, they should be able to manage. And I didn't think that that would be a trigger for readmission because pain should get better with time. So I'm not quite sure why it would be of high importance."<br><br>**Study ID 8 (consultant orthopaedic surgeon): "**I don't have experience, but just the wording, when it is says inadequate, by definition, is inadequate. So what Study ID 9 (consultant anaesthetist) was saying is like, you know, if it's adequate, it's fine. But by definition, they're saying it's inadequate here."<br><br>**Study ID 4 (consultant orthopaedic surgeon): "**I guess in my experience, poor pain control is one of the reasons patients get readmitted. You should have you should have data on the common reasons for readmission."
Surgical factors: prolonged/complex/difficult surgery, surgical misadventure	**Study ID 9 (consultant anaesthetist): "**For me, I put moderate, sorry put high rather than moderate, but that's my reading of a statement that has mixed things in it. Surgical misadventure is dramatically different from just a difficult citing of the prosthesis. So I put more, like if I put weight on just difficult surgery, I would put it as moderate. But if I put more weighting on a surgical misadventure, then I would put towards high, which is what I did."<br><br>**Study ID 4 (consultant orthopaedic surgeon): "**So I guess, you know, in my experience, long, difficult surgery is more likely to be associated with complications and therefore readmission. And so I regarded that as of high importance."
Poor understanding of disease and post-operative course	**Study ID 3 (consultant anaesthetist): "**Just because that is one of the big principles of fast-track programs or enhanced recovery after surgery programs, with patients having a good understanding and a good understanding of what's going to happen to them on the day of surgery and for the next few days. And how that translates to

	going home and staying home and what to expect at home. And to understand what's normal and what's not normal."<br><br>**Study ID 4 (consultant orthopaedic surgeon): "**I think poor understanding disease, I agree with [Study ID 3 (consultant anaesthetist)] that it is not that it's not important, but in terms of causing a readmission, I think that it's probably a fairly low importance in terms of causes of readmission. There are other things more likely."
Poor compliance with rehabilitation	**Study ID 10 (orthopaedic registrar): "**This may be that I haven't, thanks to COVID, seen enough knee arthroplasty at St. Vincent's, but certainly at other places, I guess, poor compliance with rehab is just as a risk factor of stiffness and readmission for secondary procedures for, you know, within 30-day early manipulation. So yeah, that's why I had it. Look, this- it may not be a St Vincent's risk factor as much, because that may be less the protocol, but certainly a wider Australia that's how I'd read it."<br><br>**Study ID 7 (orthopaedic registrar):** "That's how I read it as well as same as [Study ID 10 (orthopaedic registrar)]. So that's why I put it as moderate importance. But just thinking now, usually I guess we do our MUAs at about six weeks when we follow the patients up, so it may not be a risk factor, necessarily for 30-day readmission."
Resilience	**Study ID 9 (consultant anaesthetist): "**I'm just not aware of any resilience scale. So I know it's a commonly understood way of raising children these days, but is there a patient relevant resilience scale that we can use? I was just unsure of that, so that's why put as low importance."<br><br>**Study ID 4 (consultant orthopaedic surgeon): "**Yeah, I guess I thought it interacted with some of the other things like pain catastrophizing. Those sort of patients are probably of low resilience."<br><br>**Study ID 10 (orthopaedic registrar): "**Yeah, my understanding is that there is a way of this- I can't remember what it's called, I was looking at it last night, a brief resiliency scale, I think, which is used in research as a way of looking at outcome in joint arthroplasty, and it's relatively highly associated with poorer outcomes. So then I had it as a risk factor, therefore, for those, poor outcomes are often readmissions as well."
Previous readmission (e.g. with prior contralateral TKA)	**Study ID 4 (consultant orthopaedic surgeon): "**Yeah, I didn't feel that just because of patients previously had a readmission, that that would necessarily make them at higher risk. I guess I thought that the things that have caused their readmission have been identified and would then be managed better for second procedure."<br><br>**Study ID 4 (consultant orthopaedic surgeon): "**Yeah I don't know about reduce their risk, but not mean that they're at increased risk of readmission. But there might be data out there that says the other one I may not be right about."

Haemoglobin - pre-op and on discharge	**Study ID 7 (orthopaedic registrar): "**I had it as low importance because I think that obviously it would impact their rehab and their acute inpatient stay, but I think we're pretty good at- we check it, the haemoglobin pre-op and then day one post-op, and we're pretty good at monitoring that and transfusing as necessary. So I wouldn't have thought that on discharge necessarily, you know, that it would be low enough to require- that they'd have symptomatic hypotension and things that require readmission for that."<br><br>**Study ID 8 (consultant orthopaedic surgeon): "**So I agree with that, and I think, you know, don't treat a number. If they're feeling well and are discharged even with a slightly lower haemoglobin. I don't think that's a problem. So I think it's more how they feel than the number, per se."<br><br>**Study ID 4 (consultant orthopaedic surgeon): "**I've always thought that a haemoglobin drop and transfusion was associated with a higher rate of complications. And so it's- so transfusion is associated with the higher rate of infection, that's most definite. And so- and then a significant haemoglobin drop would be, I suspect, more likely to lead to other complications such as cardiac. Maybe I'm confusing inpatient complications versus readmission, but certainly infection is a cause for readmission, so that is higher in patients who drop their haemoglobin."<br><br>**Study ID 10 (orthopaedic registrar): "**Yeah I had low. I mean, I guess it's one of those ones that's line ball, but I think there is certainly a subgroup of people who it clearly is important for. But I think as a marker itself, haemoglobin is pretty poor. I think it's been shown that day one post-op bloods for this reason doesn't actually- those total numbers don't affect 30 day outcomes. So I think as a marker in and of itself, it probably isn't a great predictive tool on who's going to get those. But there's clearly a subgroup who get symptomatic and then have those problems that it would be, but I just don't think it's a great marker for it."
Delayed surgery	**Study ID 8 (consultant orthopaedic surgeon): "**Does it include not being called for?"
Inadequate nursing ratios	No reasons given
Threshold for readmission - e.g. the specific ED, or whether there is a junior registrar reviewing the patient	**Study ID 8 (consultant orthopaedic surgeon): "**So I guess if the threshold is lower for readmission then the threshold is lower for readmission. I think you- it's late at night, you know, junior people in ED, junior people reviewing the patient. They're all more likely just to admit to sort patients out as opposed to having the knowledge to quickly sort it out in ED. So that's my reasoning."<br><br>**Study ID 7 (orthopaedic registrar): "**Yeah, I just changed my answer. I put low, but I think it's moderate. I think, yeah, more junior, like specifically, I would know that if it was late, I thought that probably I would have a low threshold to call my consultant because you're more junior. But if you say it's late at night, overnight,

	quite often I just meet someone to sort them out and do things in the morning. So that's why I chose my answer."<br><br>**Study ID 4 (consultant orthopaedic surgeon): "**Yeah, [Study ID 7 (orthopaedic registrar)] just persuaded me that that probably, I had it down as low before, and I've just nudged it up to moderate. I remember being there."<br><br>**Study ID 10 (orthopaedic registrar): "**I went from moderate to high. I think, through all of those factors, that they probably do become fairly important in terms of the overall risk of a patient being admitted, with all of the policies through ED, particularly four-hour rules and everything that we have in place now. And then the people who are around seeing how quickly they can turn around a patient. I think it probably is a fairly important risk factor of whether or not they're going to come back in through or whether they can be sorted out quickly and easily and sent on their way."
Multi-joint disease	**Study ID 7 (orthopaedic registrar):** I picked moderate importance because I thought it was a similar question to the catastrophizing patient and, you know, inadequate pain management for that patient, but I don't think it's necessary. I think it depends on how you manage those patients while they're inpatients and educate as well. I don't think it's necessarily like a really, really high association, but I thought it was moderate.
Osteoporosis	No reasons given
Ethnic minorities. In the US - black population. In Australia, Aboriginal and Torres Strait Islander Peoples. Refugees. Homelessness	**Study ID 8 (consultant orthopaedic surgeon): "**I think it was just like the one question we had before is that they're probably higher risk, but they're less likely to represent, for example, just thinking about the homeless patients we have. They kind of disappear and never come back, even though they're in a much higher risk of things going wrong. They just tend not to come back. So it evens out."<br><br>**Study ID 9 (consultant anaesthetist): "**I also found the refugees is a- I think you have to be a bit more specific, I find. A recent refugee or some of the refugees that have been here for a long time, they may be the same with the native population in terms of the social determinants of health. There's also survivor bias with refugees, so they tend to be healthier because- if you're really sick, you don't you don't you're not able to migrate or survive the refugee experience, so it's a mixed bag here, all these groups together. Yeah, not entirely clear."
IVDU = intravenous drug use; TKA = total knee arthroplasty; ED = emergency department; MUA = manipulation under anaesthesia	

Table S7: Consensus calculations for focus group

Study sample	Krippendorff's alpha (95% confidence interval)	
	**Initial vote**	**Final vote**
All participants	0.1870 (-0.0002 to 0.3866)	0.1633 (-0.0302 to 0.4070)
0.5 threshold, high importance	-0.0325 (-0.1531 to 0.1291)	-0.0067 (-0.1486 to 0.2222)
0.5 threshold, moderate importance	-0.0427 (-0.1610 to 0.1188)	-0.0001 (-0.1006 to 0.1000)
0.5 threshold, low importance	-0.0056 (-0.1154 to 0.0839)	-0.0600 (-0.1429 to 0.0431)
2/3 threshold, high importance	-0.0467 (-0.1575 to 0.1072)	0.0286 (-0.1333 to 0.1500)
2/3 threshold, moderate importance	-0.0208 (-0.1471 to 0.1630)	-0.0729 (-0.1468 to 0.0155)
2/3 threshold, low importance	-0.0451 (-0.1565 to 0.0692)	-0.0600 (-0.1429 to 0.0431)
Consultant anaesthetists	0.4242 (-0.0441 to 0.7497)	-0.1512 (-0.5707 to 0.2632)
Consultant orthopaedic surgeons	0.0818 (-0.3742 to 0.5244)	0.2572 (-0.2210 to 0.6783)
Orthopaedic registrars	0.3511 (-0.1696 to 0.7632)	0.2759 (-0.2695 to 0.7264)
All orthopaedic participants (combined category: consultants and registrars)	0.1610 (-0.0902 to 0.4292)	0.2331 (-0.0004 to 0.4966)
*threshold refers to the proportion of votes in that category, e.g. '0.5 threshold, high importance' means the calculation for Krippendorff's alpha was restricted to risk factors for which at least half the votes were in the 'high importance' category		

Figure S1: Counts of votes - Focus group initial votes

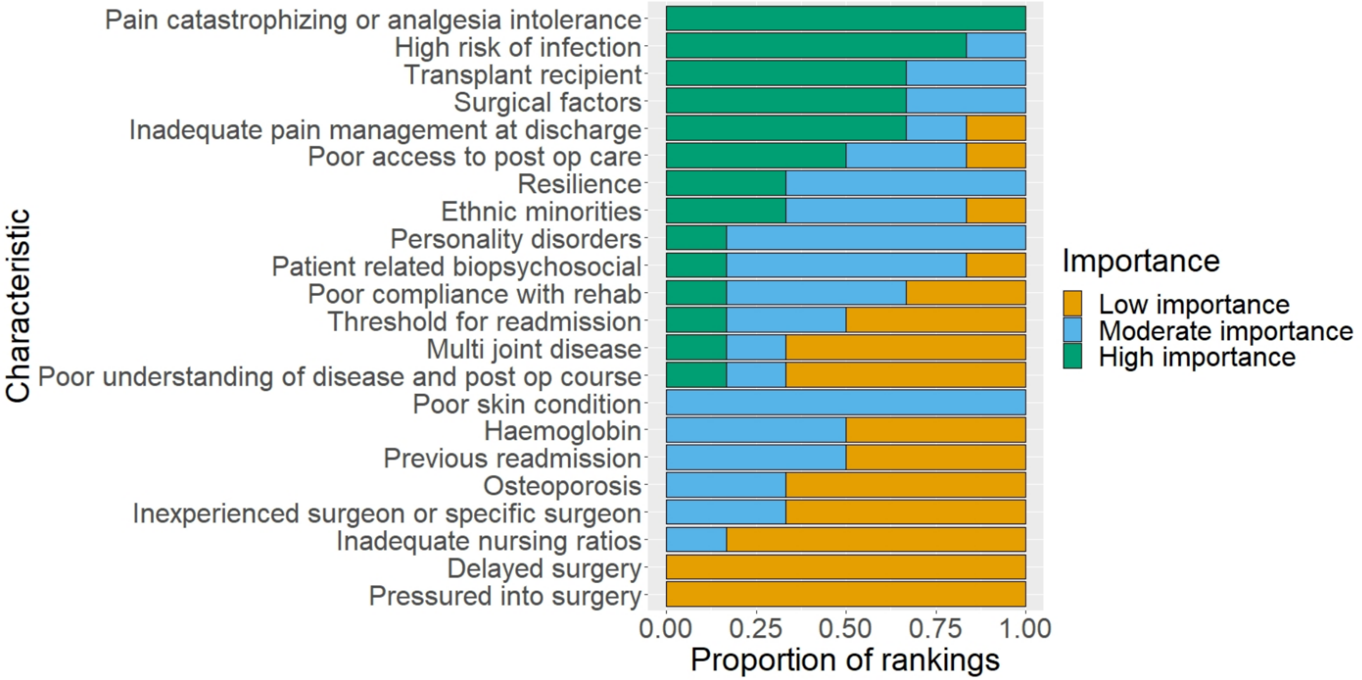

Figure S2: Focus group thematic analysis coding tree

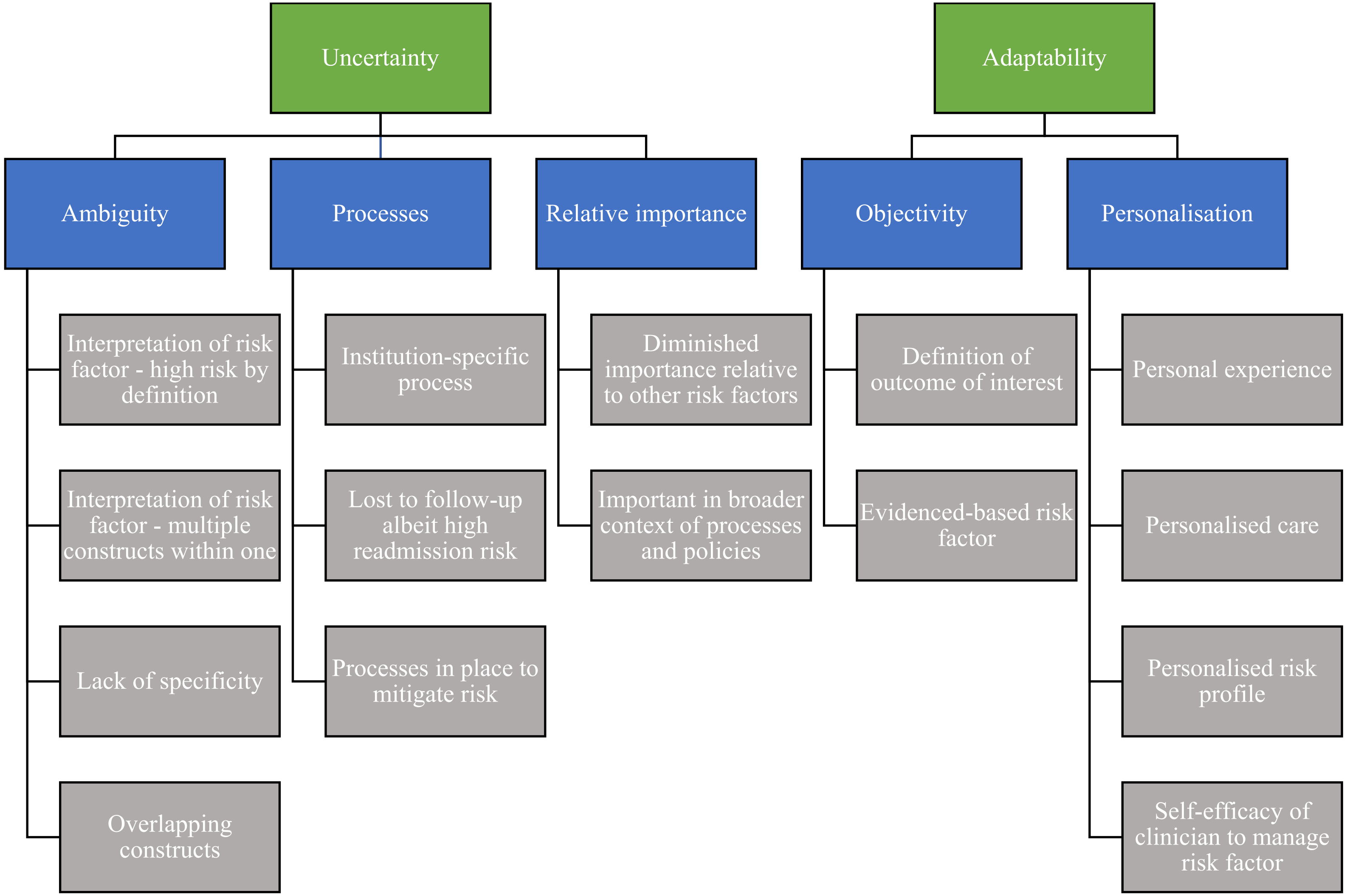

Top layer (green) = themes; middle layer (blue) = code categories; bottom layer (grey) = codes
